# Hexagonal Boron Nitride Monolayers as Protective Barriers for Graphene during Thermal Annealing

**DOI:** 10.1021/acsanm.5c05267

**Published:** 2026-03-05

**Authors:** Vladimir Calvi, Matthew D. Barnes, Dominique J. Wehenkel, Michele Buscema, Irene M. N. Groot, Richard van Rijn

**Affiliations:** † Applied Nanolayers B.V., Feldmanweg 17, 2628CT Delft, ZH, Netherlands; ‡ Leiden Institute of Chemistry, 4496 Leiden University, Einsteinweg 55, 2333 CC Leiden, Netherlands

**Keywords:** graphene, hexagonal boron nitride, thermal annealing, protective coatings, surface chemistry, 2D materials, heterostructures

## Abstract

In this study, we investigate the behavior of CVD-grown monolayer hexagonal boron nitride (h-BN) as a protective barrier on top of CVD-grown monolayer graphene during the removal of poly­(methyl methacrylate) (PMMA) residues by thermal annealing in air. We use a combination of Raman spectroscopy, optical microscopy, and atomic force microscopy to measure the damage of graphene and the efficacy of air annealing as a cleaning method. Our experimental findings demonstrate the effectiveness of h-BN as a protective barrier against graphene damage at temperatures up to 500 °C compared with bare graphene.

## Graphical abstract

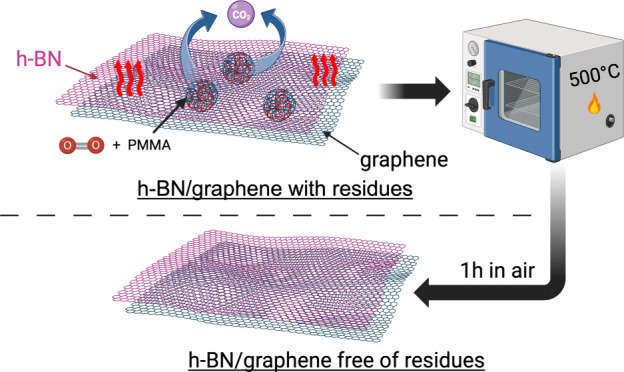

## 1 Introduction

Graphene has attracted significant attention due to its exceptional electronic, optical, thermal, and mechanical properties and its wide-ranging potential for applications.^1–3^ However, the presence of surface contaminants, such as polymer residues, poses challenges in utilizing graphene for various purposes.^4^ In the processing of graphene grown by chemical vapor deposition (CVD), either during transfer and/or during lithography, polymers are applied to the graphene surface.^5^ Polymeric residues remain present, even after thorough solvent cleaning. Thermal annealing in either air, an inert atmosphere, or vacuum is often used to further reduce the amount of residues on the graphene surface. However, the graphene gets damaged in the process as removing carbonaceous residues from a layer of carbon (graphene) with 100% selectivity is a hard problem.^6–8^ Therefore, the development of protective strategies to prevent damage and preserve the integrity of graphene during annealing is potentially useful. Hexagonal boron nitride (h-BN) is a promising protective layer for graphene during these processes. With its high chemical stability and resistance to high temperatures, h-BN can act as a passivation barrier against the diffusion of oxygen and other reactive species toward the underlying substrate.^9–15^ This is well-known for bulk applications, for example, heating elements where pyrolytic graphite heaters are protected from oxidation by pyrolytic boron nitride. Here, we aim to test if similar protective effects can also be achieved using monolayer graphene and monolayer h-BN, by inspecting the protective capabilities of h-BN during the graphene annealing processes, and its influence on the properties of graphene is crucial for developing reliable cleaning methods and strategies for graphene-based devices and materials.^16,17^ This study uses Raman spectroscopy and atomic force microscopy (AFM) to investigate the variation in graphene’s damage and its cleanliness during the annealing process at different temperatures in air while being protected by a layer of h-BN.

## 2 Methods

Monolayer graphene was grown on a Cu(111)/sapphire epitaxial substrate and transferred to a Si/SiO_2_ substrate using the electrochemical delamination method (ECD).^18^ We used commercially available CVD-grown monolayer h-BN on a copper (Cu) foil produced by Grolltex.

The transfer was done in 3 steps, which are illustrated in Figure 1. We applied two electrochemical delamination steps. In the first delamination, we applied a protective carrier stack consisting of thermal release tape (TRT) and a 600-nm-thick PMMA layer on top of the h-BN/Cu foil. The TRT/PMMA/h-BN stack was then delaminated from the Cu foil by using the ECD method. The TRT/PMMA/h-BN stack was then bonded to the graphene/Cu/sapphire substrate. In a second electrochemical delamination, the TRT/PMMA/h-BN/graphene stack was produced after the delamination of the graphene from the Cu/sapphire substrate.

**Figure 1:**
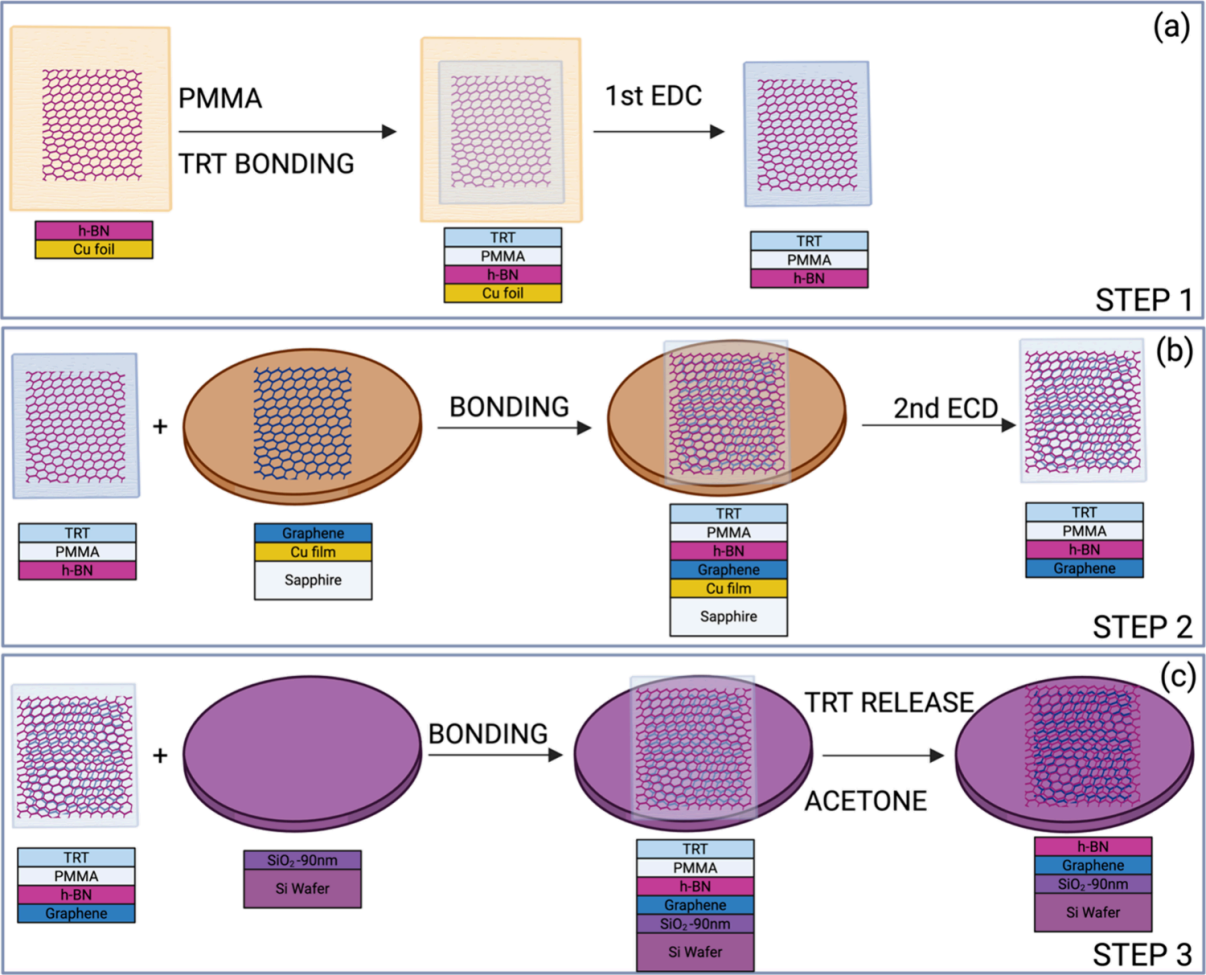
Schematic illustration of the transfer process proposed in 3 steps. (a) STEP 1: Cu foil/h-BN being transferred to the TRT/PMMA/h-BN stack. (b) STEP 2: The TRT/PMMA/h-BN stack is bonded to the sapphire/Cu film/graphene and subsequently delaminated electrochemically. (c) STEP 3: The TRT/PMMA/h-BN/graphene stack is bonded to the Si/SiO_2_ substrate.

Subsequently, the TRT/PMMA/h-BN/graphene stack was bonded to 90-nm-thick SiO_2_ and a 100 mm in diameter SiO_2_/Si wafer substrate. The TRT was released at 130 °C, and most of the PMMA was removed by dissolving it in an acetone bath in two rounds with a duration of 30 min each.^19^ The procedure for preparation of all groups of samples was as follows: bare graphene, h-BN/graphene, and monolayer h-BN were transferred to separate SiO_2_/Si substrates using the same ECD conditions. Post transfer, the 3 wafers were separately cleaved with a diamond pen into 15 samples (5 from each wafer), each of them at least 10 mm × 10 mm in size.

To characterize the transferred stack before and after the annealing, we have used Raman spectroscopy and optical microscopy to identify the possible defects formed during the transfer procedure.^20^ Raman spectroscopy was performed using a home-built system with a 532 nm laser, with the grating at 600 lines/mm, and a CCD detector model iDus 420. For each sample, we collected 1000 individual spectra at 2s integration time on a square grid 10 mm × 10 mm in size. All spectra were referenced to the Si peak at 520 cm^–1^. We used a pseudovoigt function to fit the peaks’ intensities and used the *I*_D_/*I*_G_ ratio as a main reference parameter for this study. The final distribution of *I*_D_/*I*_G_ ratios among different samples was compared by using their mean values among 1000 samplings and coupled with the relative standard deviations. In order to analyze the graphenes’ doping and strain properties, the G and 2D band frequencies were extracted from the data set and plotted with respect to the well-known reference model.^21^ Atomic force microscopy (AFM) measurements were performed on a Bruker Dimension FastScan, and the tip utilized was a ScanAsyst-Air. The root-mean-square roughness (RMS) was extracted from 3 AFM images per each sample at randomly selected areas of size of 10 μm × 10 μm. Rare residues with a measured height >20 nm were considered as abnormal debris, i.e. dust, and manually excluded from the RMS roughness calculation area in order to represent the average sample surface. Annealing was performed in a CARBOLITE AAF 1100 oven in air with a closed door. We performed 5 annealing rounds at different temperatures. To ensure reproducibility and exclude the influence of the heating ramp, all annealing experiments were performed by inserting the samples only after the oven had reached a stable set point temperature. This approach eliminates differences in ramp-up time among temperature steps, which could otherwise expose samples to different durations within the PMMA-degradation temperature window (220–300 °C). This preheating strategy ensures consistent exposure conditions for all samples. A fixed annealing time of 60 min was chosen as a representative duration for practical cleaning conditions. Owing to the small thermal-expansion coefficient mismatch between Si/SiO_2_ and graphene (<0.5% at 500 °C), no crack formation was expected. For convenience, we will indicate bare graphene single-layer samples with (G), h-BN/graphene heterostructure samples with (BNG), and the h-BN monolayer with (BN). The Raman spectra and AFM images of the references (G1_ref_ - G5_ref_) were collected before and after annealing. We will call the annealed samples G1, G2, G3, G4, and G5, annealed at 100, 200, 300, 400, and 500 °C, respectively. The same logic was applied for the h-BN/graphene (BNG) and single-layer h-BN (BN) samples’ group. The BN group was annealed only for reference to verify the integrity of the h-BN monolayer at given temperatures. In order to guarantee the comparison among the samples, each annealing round was performed on 3 samples in parallel (G, BNG, BN).

## 3 Results and Discussion

### 3.1 Reference Set for G and BNG

The comparisons between a typical G_ref_ vs BNG_ref_ are shown in Figure 2. The initial average distribution of *I*_D_/*I*_G_ ratios is 0.016 for G_ref_ and 0.02 for BNG_ref_ as shown in Figure 2a, which indicates low disorder and the absence of defects in the graphene lattice structure for both G and BNG samples’ groups.^22^ This distribution value was taken as a wafer scale average among 5 different samples of each group; we report more detailed *I*_D_/*I*_G_ ratios of all samples in the Supporting Information, Figure S1. The average *I*_D_/*I*_G_ distribution in the h-BN/graphene heterostructure (BNG) indicates that the graphene has an *I*_D_/*I*_G_ ratio of 0.020 ± SD of 0.021, which is comparable with the *I*_D_/*I*_G_ ratio of bare graphene (G) of 0.016 ± SD of 0.043; therefore. we can assume the graphene quality defined as the absence of defects being the same for both the sample groups. Representative single Raman spectra are shown in Figure 2b for all of the sample types. Moreover, single spectra were taken of transferred single-layer h-BN in order to verify the contribution of the E_2g_ peak of h-BN. We found that the E_2g_ peak located at 1372 cm^–1^ is close to the value that has been reported for the unstrained monolayer h-BN at 1370 cm^–1^, which is different form the bulk h-BN peak position being at 1366 cm^–1^.^18,23,24^ Meanwhile, the D peak of graphene is expected to appear at 1350 cm^–1^, and these two peaks may overlap if the D peak is wide, especially once the disorder in the graphene is increased due to the formation of defects. The E_2g_ peak can contribute to the D band in our fitting procedure.^25^ However, the intensity of the E_2g_ peak is low compared to that of the D peak of graphene. Therefore, the contribution of E_2g_ to the D peak can be neglected due to its low numerical contribution. Optical images and AFM measurements were acquired from randomly selected 10 μm × 10 μm regions distributed across the 10 × 10 mm sample chips. Figure 2c–f shows characteristics of typical CVD graphene covered with PMMA residues as commonly described in several studies.^19,26^ The RMS roughness of the G_ref_ sample is 1153 ± SD 110 pm on average, which is lower with respect to the 1597 ± SD 76 pm of the BNG_ref_ sample. This may be due to trapping of organic molecules at the interface between h-BN and graphene during the transfer process as shown in other studies.^17^ Triangular shapes were observed on the surface of the BNG samples’ group. They are likely due to the partial growth of the second layer of h-BN produced during the synthesis. This is confirmed by the fact that they are also present on the BN sample group (see the Supporting Information, Figure S2). Overall, all of the samples look consistent and uniform at a large scale under the optical microscope, AFM, and Raman spectroscopy.

**Figure 2:**
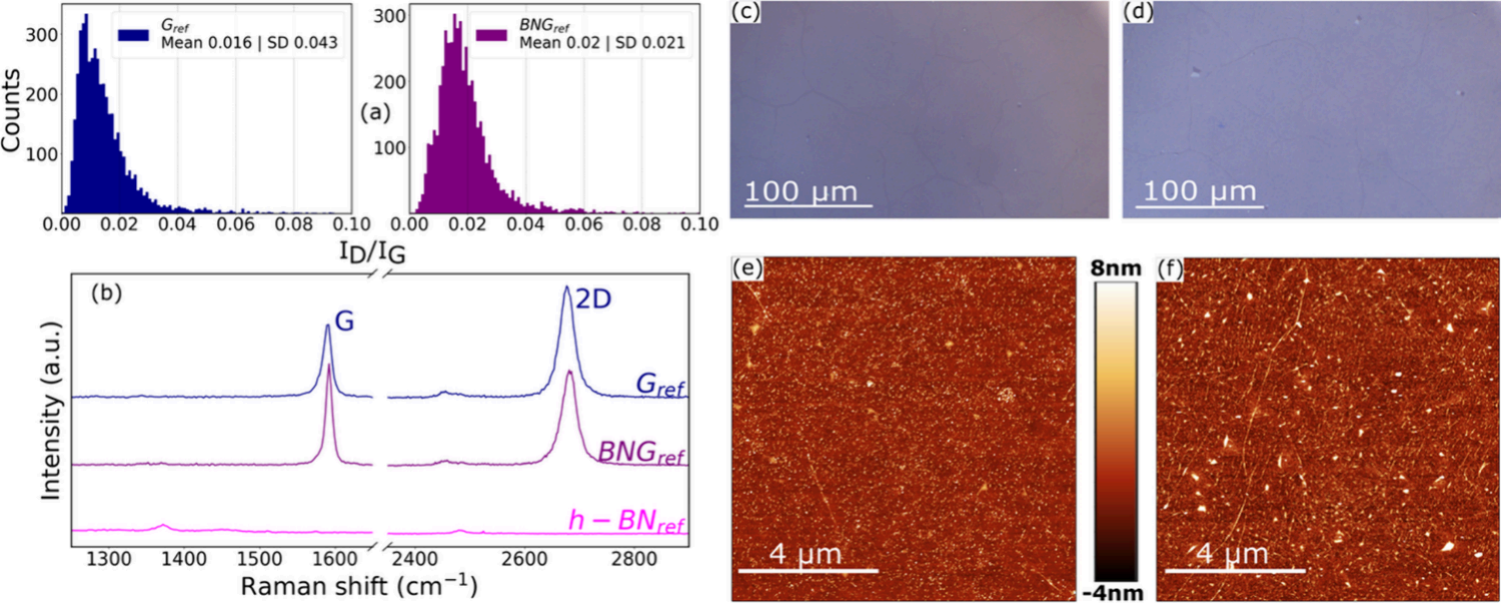
Reference samples G_ref_ and BNG_ref_. (a) *I*_D_/*I*_G_ distribution of bare graphene (G) and the h-BN/graphene stack (BNG). (b) Single Raman spectra of the reference samples: bare graphene with 2s integration time, h-BN/graphene with 2s integration time, and bare h-BN with 15s integration time. (c, d) Optical images of graphene and graphene/h-BN, respectively. (e, f) AFM images of graphene and graphene/h-BN, respectively.

### 3.2 Postannealing AFM Measurements

In order to create the representative data set, we have performed AFM scans at randomly selected areas, considering the large dimension of the samples. Random selection of regions minimizes operator bias and ensures that the reported RMS roughness values and their standard deviations are statistically representative of the overall sample surface morphology. AFM data in Figure 3 show the typical surface morphologies of the samples after the annealing processes. The bare graphene samples (G) show that in the milder annealing temperatures samples G1 and G2 do not produce a significant decrease in roughness with respect to G_ref_. The overview can be seen in the Supporting Information, Table S1. Investigation of the G1 sample shows the RMS roughness to be 1062 ± SD 114 pm on average, with respect to the 1153 ± SD 110 pm of G_ref_. The G2 sample shows a roughness up to 968 ± SD 150 pm on average. A significant decrease in roughness was observed after higher annealing temperatures for samples G3 = 493 ± SD 91 pm, G4 = 371 ± SD 17 pm, and G5 = 401 ± SD 77 pm. The surface appears to be cleaner and smoother on the latter samples. Overall, the biggest decrease in RMS roughness we could detect was from an average of 1062 pm down to 371 pm in the sample G4, which indicates a decrease of roughly 60% with respect to the reference. On the samples G3 and G4, we could observe cracks on the graphene surface. In the case of the G5 sample, no cracks were observed at all.

**Figure 3:**
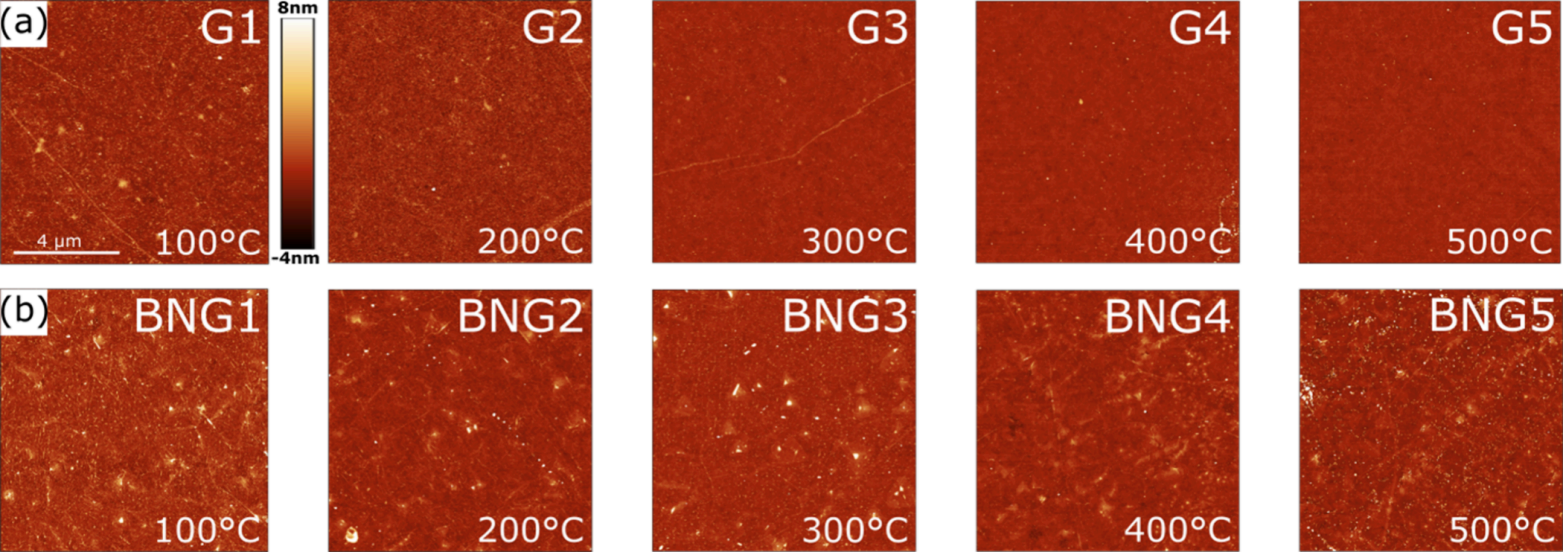
AFM images taken at randomly selected areas. (a) Samples G1–G5 with bare graphene. (b) Samples BNG1–BNG5 with the h-BN/graphene heterostack.

The samples of the BNG group shown in Figure 3b become smoother after annealing as the temperatures increase. The BNG_ref_ RMS roughness along 15 different scans (3 per sample) was 1597 ± SD 76 pm on average. Samples BNG1 = 938 ± SD 159 pm and BNG2 = 1070 ± SD 101 pm show a decrease in roughness values with respect to the reference. The samples BNG3–5 show an RMS roughness of BNG3 = 1182 ± SD 59 pm, BNG4 = 647 ± SD 101 pm, and BNG5 = 1368 ± SD 224 pm. We observed a decrease in roughness with respect to the reference for samples BNG1–BNG5, which is in line with what we observed for the G group samples. The maximum decrease observed was by roughly 60% in the sample BNG4 with respect to the reference.

### 3.3 Postannealing Raman Measurements

We show in Figure 4 typical Raman spectra of all of the annealed samples together with histograms of the *I*_D_/*I*_G_ ratios of 1000 spectra for each sample. In Figure 4a, for the bare graphene samples, we can see that the D peak only starts to increase significantly in intensity and produces an *I*_D_/*I*_G_ ratio of 0.063 ± SD 0.017 in sample G3 (300 °C). It is worth mentioning the decrease in the 2D band intensity in the same sample and the next ones (G4–G5), which indicates that the graphene structure is starting to get damaged.^20^ This damage is expressed as an increase in the *I*_D_/*I*_G_ ratio and becomes more pronounced at higher temperatures (G4). At 500 °C, we see the 2D peak almost completely disappearing, along with a decrease of the absolute G band intensity, which indicates that we are likely losing the material.^27^ Since in sample G5 we could also not identify cracks or any other feature of typical graphene in the AFM measurements, we speculate that graphene has almost completely reacted with atmospheric oxygen at 500 °C and what was left was just traces of amorphous carbon.

**Figure 4:**
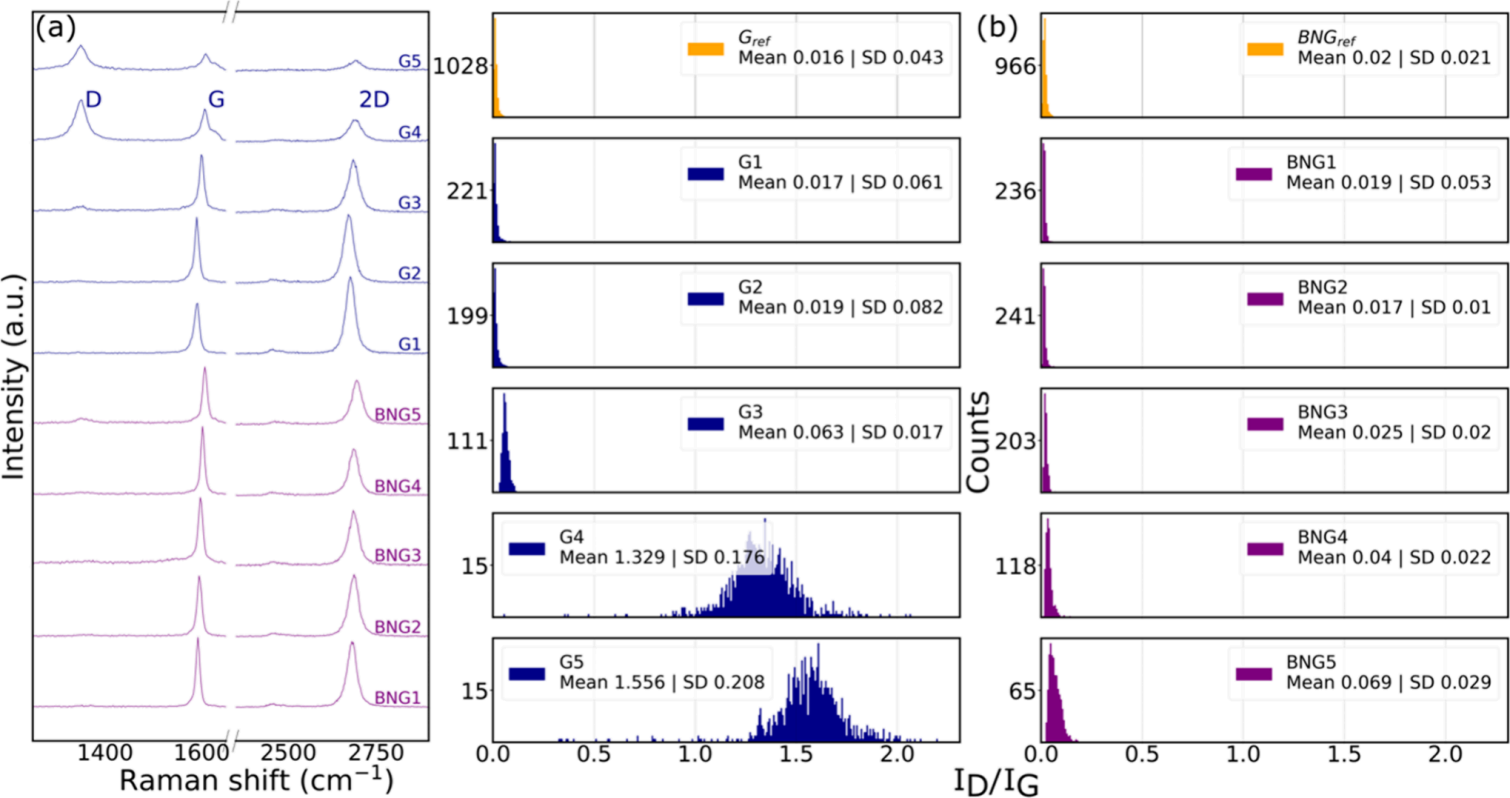
(a) Typical single Raman spectra of all samples, blue: bare graphene samples, pink: h-BN/graphene stack samples. (b) Statistical distribution of the *I*_D_/*I*_G_ intensity ratios from collected spectra per sample.

In Figure 4b, the samples G_ref_ and BNG_ref_ (in yellow) represent the reference distributions of the 2 groups of samples. In the case of samples G1 and G2, the *I*_D_/*I*_G_ ratio varies from 0.016 ± SD 0.013 to 0.017 ± SD 0.061, which is not a significant variation. We observe the formation of defects in graphene as the *I*_D_/*I*_G_ ratio starts to shift to higher values, as shown in sample G3. This is in line with expected degradation temperature of PMMA being above 200 °C, which opens to the possibility of graphene being exposed with higher surface area to air and react with atmospheric oxygen at given temperatures.^28^ A similar, more pronounced shift in the distribution is observed for sample G4, indicating that graphene undergoes progressive damage as the annealing temperature increases in each experiment. The *I*_D_/*I*_G_ ratio of G5 increases to 1.556 ± SD 0.208, indicating severe structural damages.^29^

Meanwhile, the BNG group shows that the *I*_D_/*I*_G_ ratio does not vary significantly, regardless of all of the annealing conditions that have been tested. The samples BNG1 and BNG2 have a similar *I*_D_/*I*_G_ distribution ratio. The samples BNG3 and BNG4 have a slight change in the mean value from the reference of 0.02 ± SD 0.021 to 0.025 ± SD 0.02 and 0.04 ± SD 0.022, which may indicate an increase in the *I*_D_ band. For the sample BNG5, we observed a slight change in the *I*_D_/*I*_G_ distribution ratio up to 0.069 ± SD 0.029, but this is not as significant as it is for the graphene G3–G5 samples. The comparison between BNG5 and G5 shows clearly the effectiveness of the protection of graphene by h-BN. Overall, Raman spectroscopy confirms that the 5 samples of the stacked h-BN/graphene do not show a significant variation of the *I*_D_/*I*_G_ ratio upon annealing and therefore in graphene’s quality as is shown for bare graphene samples.

### 3.4 Doping and Strain

We performed detailed analysis of the frequencies ω_G_ and ω_2D_ of each spectrum in order to extract properties such as doping (*n*) and strain (ε). The Raman shift of the G and 2D band frequencies with respect to the reference indicates how those properties change when graphene is placed at different conditions.^30–33^ We present the distribution of the band frequencies of G vs 2D in Figure 5 with their relative mean values and standard deviations. In this study, we compared our data set to the known model reported in the literature.^21^ The point of zero-doping, zero-strain is located at (1581.6 ± 0.2, 2676.9 ± 0.7) (cm^–1^). We compared doping and strain variations in our annealed samples with respect to those of our G_ref_ and HBG_ref_.

**Figure 5:**
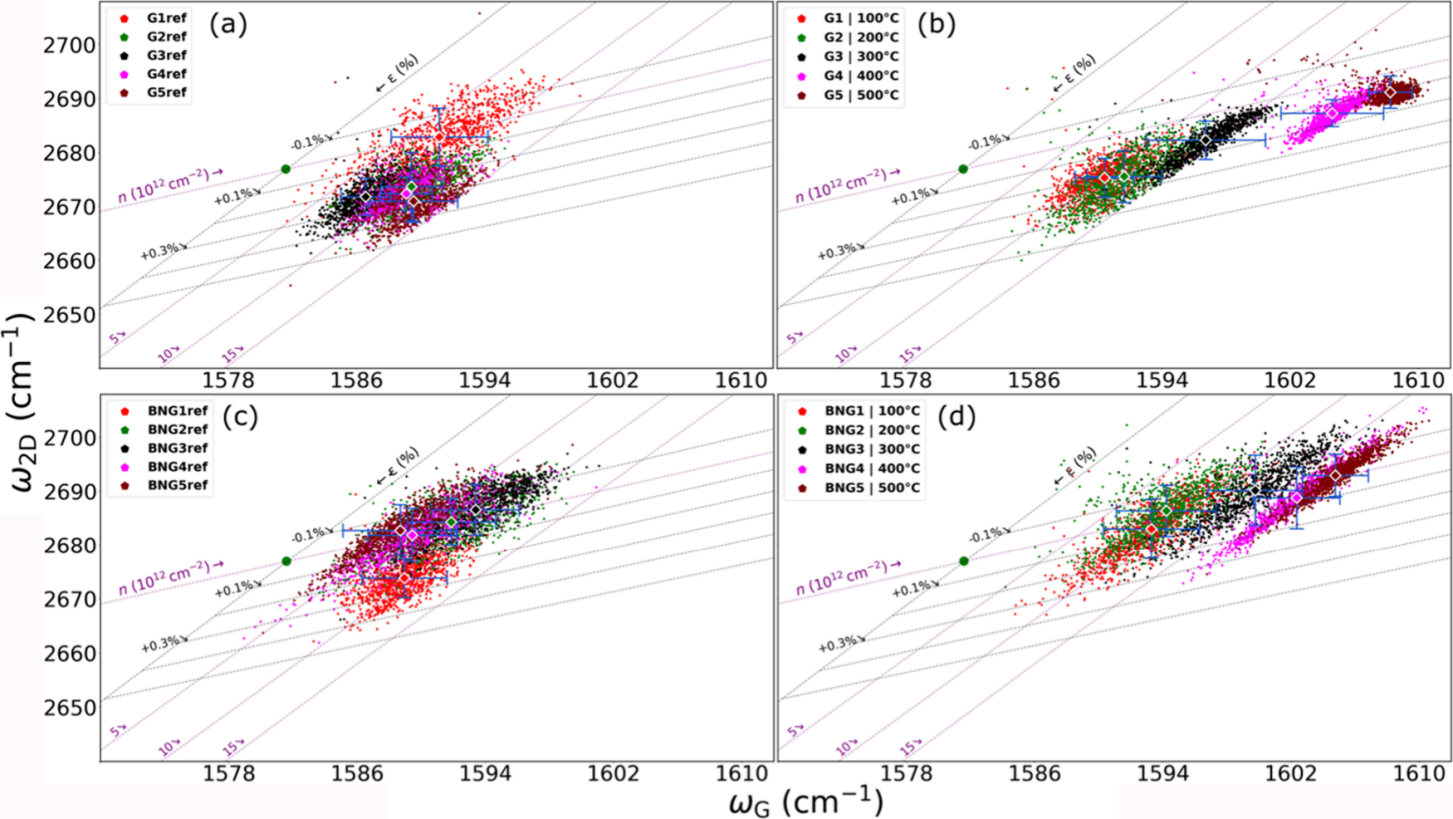
(a, b) Doping (*n*) and strain (ε) for graphene sample references and annealed samples. (c, d) *n* and ε of h-BN/graphene samples. The green dot indicates the zero-doping, zero-strain point reported from the literature.

The bare graphene group is shown in Figure 5a,b, which shows the behavior of the references G_(1–5)ref_ vs annealed group of same samples. Within the G_ref_ data set group, there is a slight doping and strain variation on the given batch of graphene. The strain of G1_ref_–G5_ref_ samples is located in the range of 0% < ε < 0.3%, and the relative doping is 5 × 10^12^ cm^–2^ < *n* < 15 × 10^12^ cm^–2^. After annealing, the strain resulted to be for samples G1–G2 ε = 0.2% and for samples G3–G5 = 0.1% < ε < 0.2%. The doping values are 10 × 10^12^ cm^–2^ < *n* < 15 × 10^12^ cm^–2^ for samples G1–G3 and >15 × 10^12^ cm^–2^ for samples G4 and G5.

From Figure 5c,d, we see that the BNG_(1–5)ref_ samples have an inherent slight variation in doping and strain. The strain values are in the range of −0.1% < ε < 0.2%. Doping was measured to be 5 × 10^12^ cm^–2^ < *n* < 10 × 10^12^ cm^–2^). For the annealed BNG samples, we observe that strain is reduced to the range of −0.1% < ε < 0.1%, which is in line with our expectations that the graphene shows reduction of the strain due to annealing.^34^ The doping values for samples BNG1–BNG3 are 10 × 10^12^ cm^–2^ < *n* < 15 × 10^12^ cm^–2^ and >15 × 10^12^ cm^–2^ for samples annealed at higher temperatures such as BNG4 and BNG5.

### 3.5 Summary

In our study, we demonstrate a cleaning process for the h-BN/graphene heterostack to remove polymeric residues used during transfer. These findings are different from similar studies where the surface roughness after annealing at 300 °C was reported to be 190 pm vs 1182 ± SD 59 pm that we could obverse in our samples.^18,35–37^ We speculate that this may be due to the different transfer procedure. Our findings demonstrate that annealing bare graphene above 300–500 °C for 1h in air produces significant defect formation as shown by the increase of the *I*_D_/*I*_G_ ratio. In contrast, for the h-BN-coated graphene samples, the *I*_D_/*I*_G_ does not move in all tested temperature ranges, suggesting that monolayer h-BN can act as a protective passivation layer for cleaning processes. This finding is relevant in biosensing applications where the removal of surface contaminants such as PMMA improves the surface functionalization and thus the sensitivity of the sensors.^38–40^ These findings open a new challenge related to the functionalization of the h-BN surface in the h-BN/graphene heterostructure. Several studies already reported that it is possible to to do.^41,42^ Our findings offer a simple thermal protocol compatible with the post-transfer strategy to clean the surface of graphene from the undesired residues. This enables a potential of the dual-mode surface engineering: using clean graphene as an active sensing element, while h-BN on top as a scaffold for receptor interaction for biosensing devices. It is also known that a protective layer of h-BN on graphene can improve the deposition of dielectrics by ALD. Graphene itself is a notoriously difficult material to deposit dielectric materials with ALD as it has no dangling bonds.^43^ A comprehensive summary is illustrated in Figure 6. We observe that the *I*_D_/*I*_G_ distribution ratios increase significantly when bare graphene samples are annealed at ≥300 °C, but for the h-BN/graphene stack, *I*_D_/*I*_G_ remains constant. Figure 6b shows the variation of the RMS roughness on average along all of the samples of the BNG and G groups. We observe that the surface is getting smoother at higher temperatures. For the bare graphene samples, the RMS roughness changes on average from the reference value of 1153 pm down to 371 pm at 400 °C annealing temperature. In the case of stacked h-BN/graphene, the RMS roughness changes on average from 1597 pm down to 647 pm. We also observe that some of the BNG samples show an unexpected increase in roughness as we progress with increasing the annealing temperature. We speculate that this may be due to an uncontrolled amount of organic molecules trapped on the interface of h-BN/graphene. Therefore, due to this contribution, it is hard to estimate the exact variation in roughness for the BNG group reliably. However, we observe a general downtrend and decrease in roughness after the annealing.

**Figure 6:**
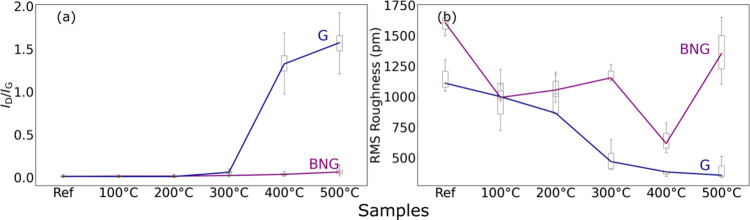
(a) Distribution of the *I*_D_/*I*_G_ ratios along all of the samples. (b) Distribution of the RMS roughness values in pm.

## 4 Conclusions

We have shown that a CVD-grown monolayer h-BN can act as a protective barrier for CVD graphene during air annealing up to 500 °C. We show that the *I*_D_/*I*_G_ ratio for bare graphene increases from 0.0160 to 1.556 upon annealing, while for h-BN-protected graphene, the *I*_D_/*I*_G_ ratio only increases to a maximum of 0.069. The onset of significant damage to the bare graphene starts between 200 and 300 °C in air. We believe that for certain applications, such as for graphene-based membranes and biosensors, the h-BN layer can be used as a protective coating upon proper functionalization.

## Supplementary Material



## Notes

*I*_D_/*I*_G_ distributions before annealing (Figure S1); AFM images of BN samples (Figure S2); and RMS roughness values of all samples (Table S1) (PDF)

## References

[1] Ferrari A. C., Meyer J. C., Scardaci V., Casiraghi C., Lazzeri M., Mauri F., Piscanec S., Jiang D., Novoselov K. S., Roth S., Geim A. K. (2006). Raman Spectrum of
Graphene and Graphene Layers. Phys. Rev. Lett..

[2] Bonaccorso F., Sun Z., Hasan T., Ferrari A. C. (2010). Graphene Photonics and Optoelectronics. Nat. Photonics.

[3] Zhong Y., Zhen Z., Zhu H. (2017). Graphene:
Fundamental Research and
Potential Applications. FlatChem..

[4] Choi W., Arslan Shehzad M., Park S., Seo Y. (2017). Influence of Removing
PMMA Residues on Surface of CVD Graphene Using a Contact-Mode Atomic
Force Microscope. RSC Adv..

[5] Borin
Barin G., Song Y., de Fátima Gimenez I., Souza Filho A. G., Barreto L. S., Kong J. (2015). Optimized Graphene
Transfer: Influence of Polymethylmethacrylate (PMMA) Layer Concentration
and Baking Time on Graphene Final Performance. Carbon.

[6] Zhuang B., Li S., Li S., Yin J. (2021). Ways to Eliminate PMMA Residues on
Graphene - Superclean Graphene. Carbon.

[7] Xiao Z., Wan Q., Durkan C. (2019). Cleaning Transferred
Graphene for Optimization of Device
Performance. Adv. Mater. Interfaces.

[8] Xie W., Weng L.-T., Ng K. M., Chan C. K., Chan C.-M. (2015). Clean Graphene
Surface through High Temperature Annealing. Carbon.

[9] Scardamaglia M., Boix V., D’Acunto G., Struzzi C., Reckinger N., Chen X., Shivayogimath A., Booth T., Knudsen J. (2021). Comparative
Study of Copper Oxidation Protection with Graphene and Hexagonal Boron
Nitride. Carbon.

[10] Surwade S. P., Li Z., Liu H. (2012). Thermal Oxidation
and Unwrinkling of Chemical Vapor
Deposition-Grown Graphene. J. Phys. Chem. C.

[11] Liu L., Ryu S., Tomasik M. R., Stolyarova E., Jung N., Hybertsen M. S., Steigerwald M. L., Brus L. E., Flynn G. W. (2008). Graphene Oxidation:
Thickness-Dependent Etching and Strong Chemical Doping. Nano Lett..

[12] Zhang K., Feng Y., Wang F., Yang Z., Wang J. (2017). Two Dimensional
Hexagonal Boron Nitride (2D-hBN): Synthesis, Properties and Applications. J. Mater. Chem. C.

[13] Wróblewska A., Dużyńska A., Judek J., Stobiński L., Żerańska K., Gertych A. P., Zdrojek M. (2017). Statistical
Analysis of the Reduction Process of Graphene Oxide Probed by Raman
Spectroscopy Mapping. J. Phys.: Condens. Matter.

[14] Krečcmarová M., Andres-Penares D., Fekete L., Ashcheulov P., Molina-Sánchez A., Canet-Albiach R., Gregora I., Mortet V., Martínez-Pastor J. P., Sánchez-Royo J. F. (2019). Optical Contrast and Raman Spectroscopy
Techniques Applied to Few-Layer 2D Hexagonal Boron Nitride. Nanomaterials.

[15] Saji V. S. (2023). 2D Hexagonal
Boron Nitride (h-BN) Nanosheets in Protective Coatings: A Literature
Review. Heliyon.

[16] Jain N., Bansal T., Durcan C. A., Xu Y., Yu B. (2013). Monolayer
Graphene/Hexagonal Boron Nitride Heterostructure. Carbon.

[17] Argentero G., Mittelberger A., Reza Ahmadpour Monazam M., Cao Y., Pennycook T. J., Mangler C., Kramberger C., Kotakoski J., Geim A. K., Meyer J. C. (2017). Unraveling the 3D
Atomic Structure of a Suspended Graphene/hBN van Der Waals Heterostructure. Nano Lett..

[18] Fukamachi S., Solís-Fernández P., Kawahara K., Tanaka D., Otake T., Lin Y.-C., Suenaga K., Ago H. (2023). Large-Area
Synthesis and Transfer of Multilayer Hexagonal Boron Nitride for Enhanced
Graphene Device Arrays. Nat. Electron..

[19] Liu N., Pan Z., Fu L., Zhang C., Dai B., Liu Z. (2011). The Origin
of Wrinkles on Transferred Graphene. Nano Res..

[20] Das A., Chakraborty B., Sood A. K. (2008). Raman Spectroscopy of Graphene on
Different Substrates and Influence of Defects. Bull. Mater. Sci..

[21] Lee J. E., Ahn G., Shim J., Lee Y. S., Ryu S. (2012). Optical Separation
of Mechanical Strain from Charge Doping in Graphene. Nat. Commun..

[22] Jorio A., Ferreira E. H. M., Moutinho M. V. O., Stavale F., Achete C. A., Capaz R. B. (2010). Measuring Disorder
in Graphene with the G and D Bands. Phys. Status
Solidi B.

[23] Blundo E., Surrente A., Spirito D., Pettinari G., Yildirim T., Chavarin C. A., Baldassarre L., Felici M., Polimeni A. (2022). Vibrational Properties in Highly
Strained Hexagonal Boron Nitride Bubbles. Nano
Lett..

[24] Liu T., Premasiri K., Sui Y., Zhan X., Mustafa H. A. B., Akkus O., Zorman C. A., Gao X. P. A., Sankaran R. M. (2018). Direct,
Transfer-Free Growth of Large-Area Hexagonal Boron Nitride Films by
Plasma-Enhanced Chemical Film Conversion (PECFC) of Printable, Solution-Processed
Ammonia Borane. ACS Appl. Mater. Interfaces.

[25] Wang J., Ma F., Sun M. (2017). Graphene, Hexagonal Boron Nitride, and Their Heterostructures:
Properties and Applications. RSC Adv..

[26] Demirbaş T., Baykara M. Z. (2016). Nanoscale
Tribology of Graphene Grown by Chemical Vapor
Deposition and Transferred onto Silicon Oxide Substrates. J. Mater. Res..

[27] Eckmann A., Felten A., Mishchenko A., Britnell L., Krupke R., Novoselov K. S., Casiraghi C. (2012). Probing the Nature of Defects in
Graphene by Raman Spectroscopy. Nano Lett..

[28] Holland B.
J., Hay J. N. (2002). The Effect
of Polymerisation Conditions on the Kinetics
and Mechanisms of Thermal Degradation of PMMA. Polym. Degrad. Stab..

[29] Cançado L. G., Jorio A., Ferreira E. H. M., Stavale F., Achete C. A., Capaz R. B., Moutinho M. V. O., Lombardo A., Kulmala T. S., Ferrari A. C. (2011). Quantifying Defects
in Graphene via Raman Spectroscopy
at Different Excitation Energies. Nano Lett..

[30] Berciaud S., Ryu S., Brus L. E., Heinz T. F. (2009). Probing the Intrinsic Properties
of Exfoliated Graphene: Raman Spectroscopy of Free-Standing Monolayers. Nano Lett..

[31] Das A., Pisana S., Chakraborty B., Piscanec S., Saha S. K., Waghmare U. V., Novoselov K. S., Krishnamurthy H. R., Geim A. K., Ferrari A. C., Sood A. K. (2008). Monitoring Dopants
by Raman Scattering in an Electrochemically Top-Gated Graphene Transistor. Nat. Nanotechnol..

[32] Mueller N. S., Heeg S., Alvarez M. P., Kusch P., Wasserroth S., Clark N., Schedin F., Parthenios J., Papagelis K., Galiotis C., Kalbáč M., Vijayaraghavan A., Huebner U., Gorbachev R., Frank O., Reich S. (2018). Evaluating Arbitrary Strain Configurations
and Doping in Graphene with Raman Spectroscopy. 2D Mater..

[33] Das A., Chakraborty B., Piscanec S., Pisana S., Sood A. K., Ferrari A. C. (2009). Phonon Renormalization in Doped Bilayer Graphene. Phys. Rev. B.

[34] Kim S., Ryu S. (2016). Thickness-Dependent Native Strain in Graphene Membranes
Visualized
by Raman Spectroscopy. Carbon.

[35] Liu J., Kutty R. G., Liu Z. (2016). Controlled
Synthesis of Atomically
Layered Hexagonal Boron Nitride via Chemical Vapor Deposition. Molecules.

[36] Garcia A. G. F., Neumann M., Amet F., Williams J. R., Watanabe K., Taniguchi T., Goldhaber-Gordon D. (2012). Effective
Cleaning of Hexagonal Boron
Nitride for Graphene Devices. Nano Lett..

[37] Engels S., Terrés B., Klein F., Reichardt S., Goldsche M., Kuhlen S., Watanabe K., Taniguchi T., Stampfer C. (2014). Impact of Thermal Annealing
on Graphene Devices Encapsulated
in Hexagonal Boron Nitride. Phys. Status Solidi
B.

[38] Baruah A., Newar R., Das S., Kalita N., Nath M., Ghosh P., Chinnam S., Sarma H., Narayan M. (2024). Biomedical
Applications of Graphene-Based Nanomaterials: Recent Progress, Challenges,
and Prospects in Highly Sensitive Biosensors. Discovery Nano.

[39] Justino C. I. L., Gomes A. R., Freitas A. C., Duarte A. C., Rocha-Santos T. A. P. (2017). Graphene
Based Sensors and Biosensors. TrAC, Trends Anal.
Chem..

[40] Pirkle A., Chan J., Venugopal A., Hinojos D., Magnuson C. W., McDonnell S., Colombo L., Vogel E. M., Ruoff R. S., Wallace R. M. (2011). The Effect of Chemical Residues on the Physical and
Electrical Properties of Chemical Vapor Deposited Graphene Transferred
to SiO2. Appl. Phys. Lett..

[41] Weng Q., Wang X., Wang X., Bando Y., Golberg D. (2016). Functionalized
Hexagonal Boron Nitride Nanomaterials: Emerging Properties and Applications. Chem. Soc. Rev..

[42] Wang C., Long Y., Deng Y., Han Y., Tishkevich D., Ha M. N., Weng Q. (2024). Hexagonal Boron Nitride
Nanomaterials
for Biomedical Applications. BMEMat.

[43] Canto B., Otto M., Powell M. J., Babenko V., O’Mahony A., Knoops H. C. M., Sundaram R. S., Hofmann S., Lemme M. C., Neumaier D. (2021). Plasma-Enhanced Atomic Layer Deposition of Al2O3 on
Graphene Using Monolayer hBN as Interfacial Layer. Adv. Mater. Technol..

