# Supplementary material for: Hexagonal Boron Nitride Monolayers as Protective Barriers for Graphene during Thermal Annealing
Source: ACS Appl Nano Mater. 2026 Mar 5;9(11):4863–70. doi: 10.1021/acsanm.5c05267 (PMC13010352; doi:10.1021/acsanm.5c05267)
Supplement: Supplementary file 1 [file an5c05267_si_001.pdf]

Supporting Information

Hexagonal Boron Nitride Monolayers as Protective Barriers for Graphene During Thermal Annealing

Vladimir Calvi[1,2], Matthew D. Barnes[1], Dominique J. Wehenkel[1], Michele Buscema[1], Irene M.N. Groot[2], and Richard van Rijn[1]*

[1]Applied Nanolayers B.V., Feldmanweg 17 Delft, ZH, NL 2628CT;

[2]Leiden Institute of Chemistry, Leiden University, Einsteinweg 55, Leiden 2333 CC;

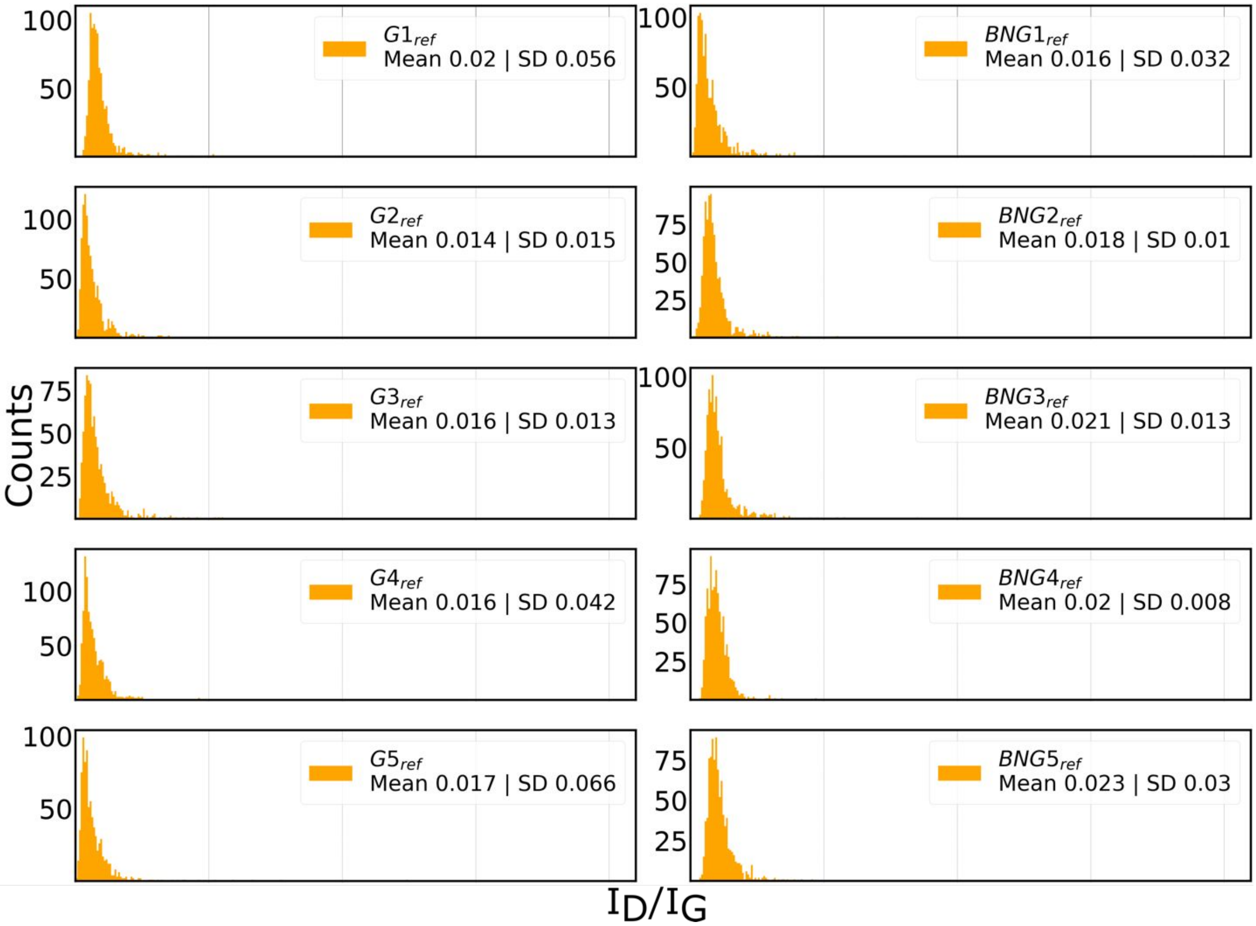

Figure S1. I_D/I_G distributions of the samples before annealing.

---

* Corresponding author.

E-mail address: r.van.rijn@appliednanolayers.com (Dr. Richard van Rijn)

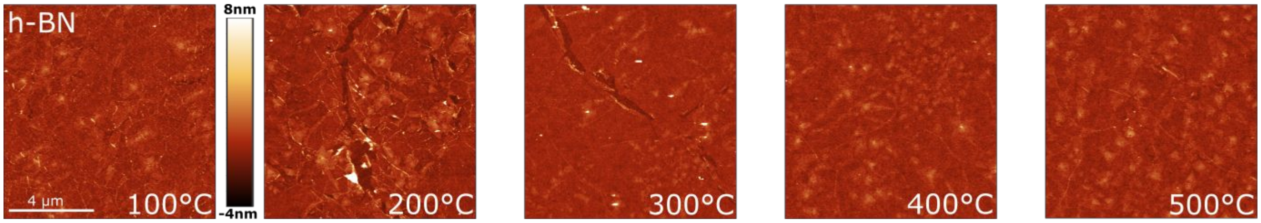

Figure S2. AFM images of BN group (h-BN single layer) samples transferred to SiO_2 and annealed in parallel with the G & BNG samples.

Table S1. Overview of the average surface roughness values on reference and annealed samples expressed in (pm).

	Ref	100°C	200°C	300°C	400°C	500°C
G	1153 +/- SD 110	1062 +/- SD 114	968 +/- SD 150	493 +/- SD 91	371 +/- SD 17	401 +/- SD 77
BNG	1597 +/- SD 76	938 +/- SD 159	1070 +/- SD 101	1182 +/- SD 59	647 +/- SD 101	1368 +/- SD 224
